# Prospective Evaluation of Blood-based and Microbiological Early Indicators of In-hospital Infectious Complications After Open Cystectomy

**DOI:** 10.1016/j.euros.2025.10.019

**Published:** 2025-11-18

**Authors:** Benedikt Ebner, Judith Hirsch, Annkathrin Holz, Lennert Eismann, Julian Hermans, Nikolaos Pyrgidis, Marc Kidess, Marie Semmler, Isabel Brinkmann, Can Aydogdu, Michael Chaloupka, Maria Apfelbeck, Andrea Katharina Lindner, Philipp Weinhold, Christian G. Stief, Yannic Volz, Gerald B. Schulz

**Affiliations:** Department of Urology, University Hospital, Ludwig-Maximilians-University, Munich, Germany

**Keywords:** Cystectomy, Interleukin-6, Procalcitonin, Wound drainage fluid, Bacterial culture, Early indicators, Postoperative infectious complications, Comprehensive Complication Index

## Abstract

**Background and objective:**

In-hospital infectious complications after cystectomy are understudied, with no reliable predictive tools. The aim of our study was to (1) comprehensively and prospectively evaluate these complications, (2) compare results between ileal conduit (IC) and ileal neobladder (NB) groups, and (3) evaluate the suitability of interleukin-6 (IL-6) and procalcitonin (PCT) levels and culture results for wound drainage fluid (WDF) as early indicators of infection.

**Methods:**

We monitored in-hospital complications among patients undergoing cystectomy and analyzed IL-6 and PCT levels and WDF cultures on the first postoperative day. Statistical analysis included logistic regression and Spearman correlation analysis. The trial was registered on ClinicalTrials.gov as NCT05153694.

**Key findings and limitations:**

From December 2021 to October 2024, 205 patients underwent open cystectomy in our department, of whom 186 consented to participate (66% IC, 34% NB). The median patient age was 71.6 yr and 80% were male. During their inpatient stay, 47% of patients developed fever and 44% received additional intravenous antibiotics. We found no significant differences between the IC and NB groups regarding postoperative fever, additional antibiotic use, positive blood cultures, the incidence of complications by Clavien-Dindo grade, Comprehensive Complication Index scores, or the incidence of wound infection or other infections. Microbial growth was detected in 13% of WDF samples; *Escherichia coli* and *Enterococcus* spp. were the most prevalent bacteria. We observed little to no correlations between IL-6 or PCT levels or WDF culture results and infectious complications.

**Conclusions and clinical implications:**

Our prospective study revealed unexpectedly high incidence of in-hospital infections among cystectomy patients, with no significant differences between IC and NB groups. IL-6 and PCT levels and WDF culture results on the first postoperative day were not suitable as early indicators of infectious complications after open cystectomy.

**Patient summary:**

We looked at infectious complications during their hospital stay for patients undergoing surgical removal of their bladder. We found no difference in the rate of infectious complications between two common surgical procedures that create a new pathway for urine to leave the body. We also found that levels of the markers interleukin-6 and procalcitonin, and bacterial culture results for wound drainage fluid were not helpful in predicting infectious complications for these patients.

## Take Home Message

Our prospective study revealed no significant differences regarding in-hospital infectious complications after cystectomy between ileal conduit and neobladder groups. A comprehensive analysis of interleukin-6, procalcitonin, and culture of wound drainage fluid revealed that they are unsuitable as early indicators for infectious complications following cystectomy.

## 1 Introduction

Cystectomy is associated with high incidence of postoperative infectious complications, with incidence rates ranging from 5% to 40% [[1], [2], [3], [4]]. However, most studies to date are limited by a retrospective design and lack of clear definitions of the term “infectious complication”. A recent systematic review by Antonelli et al. [1] that included 20 studies with a total of 55 306 patients revealed that the rate of blood infections reported after cystectomy ranged from 2% to 19%. Regarding urinary diversion, limited retrospective data suggest higher risk of long-term infectious complications for patients with an ileal neobladder (NB) in comparison to patients with an ileal conduit (IC) [[5], [6], [7], [8]]. However, there is a lack of prospective data, particularly on in-hospital complications. Level 1 evidence on the incidence of infectious complications could lead to better recommendations on the type of urinary diversion to choose, especially for vulnerable subgroups such as immunocompromised patients. Robust high-quality data are needed, as infectious complications are becoming increasingly difficult to manage because of the rise of antimicrobial resistance [9].

There is also a lack of reliable early indicators of postoperative infectious complications after cystectomy. C-Reactive protein (CRP) is one of the most widely used parameters for diagnosing infections and inflammatory processes because it is simple to measure via a low-cost test [10]. However, CRP exhibits slow kinetics and low specificity for bacterial infections [10,11], making it unsuitable as an early marker of postoperative infectious complications. In recent years, other parameters, such as interleukin-6 (IL-6) and procalcitonin (PCT), have attracted increasing interest as markers of infection [[12], [13], [14]]. IL-6 levels rise earlier than CRP levels, and thus indicate inflammation sooner, while PCT has higher specificity for bacterial infections [10,15,16]. However, the predictive value of IL-6 and PCT for infectious complications in patients after cystectomy is unclear. The role of microbial analysis of wound drainage fluid (WDF) as a diagnostic tool in surgery has been discussed [17]. In other surgical specialties, positive WDF cultures have been extensively studied and were associated with a higher incidence of postoperative complications, such as postoperative pancreatic fistula [[18], [19], [20], [21]]. While interpretation of culture WDF results after 3 d is complicated by bacterial colonization [22], early detection of noncommensal organisms in WDF (eg, on postoperative day 1 [POD1]) may help in differentiating infection from colonization and in predicting clinical outcomes. For patients undergoing cystectomy, detection of noncommensal organisms in WDF on POD1 could help in distinguishing true infection from colonization and potentially in predicting worse clinical outcomes. Samples for blood-based and microbiological early indicators such as IL-6, PCT, and WDF cultures are easily obtained and minimally invasive, and the tests are comparatively inexpensive. However, there is no evidence to date on the use of these markers in urological surgery.

The aim of this study was to (1) comprehensively and prospectively assess the incidence of in-hospital infectious complications after cystectomy, (2) compare results between IC and NB groups, and (3) evaluate the suitability of IL-6, PCT, and WDF culture results as early indicators of infection.

## 2 Patients and methods

### 2.1 Study design and patient cohort

We prospectively included patients undergoing open cystectomy for oncological or non-oncological indications between December 2021 and October 2024 at the Department of Urology, LMU University Hospital (Munich, Germany). We decided to exclusively focus on patients undergoing open cystectomy, as this approach still accounts for the vast majority of cystectomy procedures in Germany [23]. During the study period, only four patients underwent robot-assisted cystectomy in our department. These patients were not included in the present analysis. Written informed consent was obtained from each patient before enrollment. Postoperative patient care after cystectomy follows standardized protocols in our department that are based on enhanced recovery after surgery (ERAS) recommendations [24]. No routine preoperative bowel preparation is performed. As part of the hospital standard protocol, cystectomy patients receive intravenous cefuroxime for 5 d and metronidazole for 3 d. A neocystogram or conduitogram is performed after 10–14 d before removing the ureteric stents and the indwelling catheter. Prophylactic oral antibiotics before stent removal are administered at the discretion of the treating physician and depend on a urine test conducted 1–2 d before radiographic examination. For the present study, clinicopathological characteristics were prospectively collected during the hospital stay. IL-6 and PCT levels and microbial growth in WDF were analyzed on POD1. Fever was defined as body temperature ≥38.0 °C. In accordance with the standard hospital protocol, blood cultures were performed for all patients with postoperative fever. All in-hospital complications were graded using the Clavien-Dindo classification, and results were used to calculate Comprehensive Complication Index (CCI) scores (https://cci-calculator.com/). The study was conducted in accordance with the ethical standards of the Declaration of Helsinki, was approved by the university ethics committee (project number 18-059), and was registered on ClinicalTrials.gov as NCT05153694.

### 2.2 Outcome parameters

We focused on outcome parameters that are objectively measurable: the incidence of postoperative fever, antibiotic use on top of the hospital standard protocol, wound infection, other infections, positive blood cultures, complications Clavien-Dindo grade, CCI scores, and length of stay in intermediate care or the intensive care unit (IMC/ICU). The threshold for significant microbial growth in WDF or blood cultures was set at 10^3^ CFU/ml. Given the diverse and nonspecific definitions of sepsis [25], sepsis was not defined as an outcome parameter in the present study. Cystectomy patients frequently exhibit postoperative fever, significantly elevated infection markers with detectable pathogens, and temporary elevation of creatinine levels, making precise distinction from true sepsis difficult.

### 2.3 Statistical analysis

Results are reported as the median with interquartile range (IQR) for continuous variables, and as the frequency and proportion for categorical variables. A two-tailed t test for independent samples was performed for comparison of continuous variables between groups. Before every t test, a Levene test was performed to assess equality of variance. A χ^2^ test was performed for comparison of categorical variables between groups. When assumptions for a χ^2^ test were not met (eg, expected cell frequency <5), Fisher’s exact test was performed. Logistic regression analyses were performed to evaluate the association between clinicopathological characteristics, infection markers, and postoperative infectious complications. The independent variables for inclusion were chosen on the basis of clinical relevance. Spearman correlation analysis was conducted to assess the strength and direction of the association between IL-6 or PCT levels and our outcome parameters. Spearman’s rank correlation coefficients were determined. The significance level was set at 5%. All statistical analyses were conducted with DATAtab Team (DATAtab e.U., Graz, Austria).

## 3 Results

### 3.1 Clinicopathological characteristics and infection markers on POD1

**Fig. 1:**
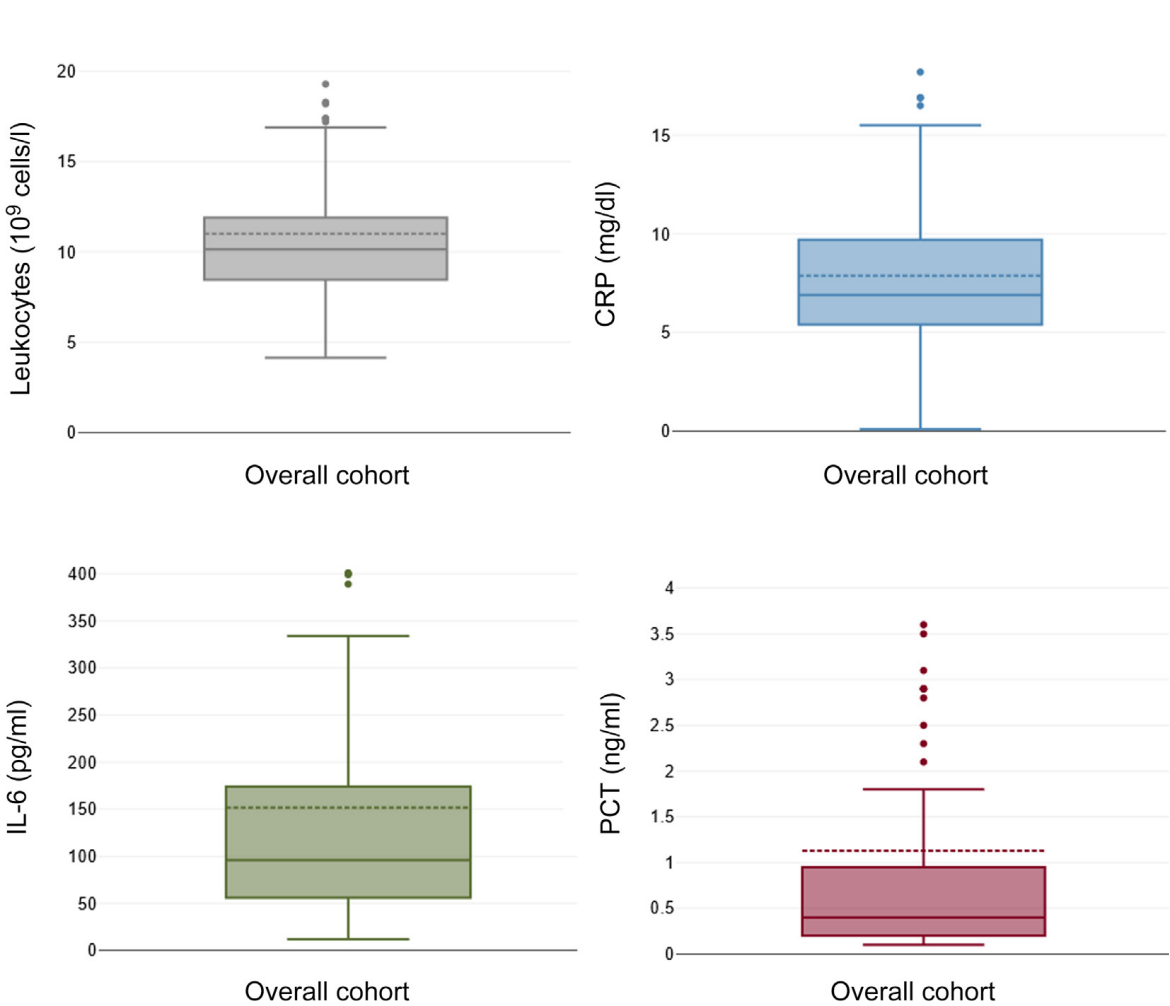
Boxplot diagram of leukocyte count, C-reactive protein (CRP), interleukin-6 (IL-6), and procalcitonin (PCT) on the first postoperative day after cystectomy. For better readability, extreme outliers are not displayed.

Between December 2021 and October 2024, 205 patients underwent open cystectomy in our department. Of these, 186 gave written consent to participate in the study and were enrolled. For urinary diversion, 122 patients (66%) received an IC and 64 patients (34%) an NB. The median age was 71.6 yr, and 80% of the patients were male. The median body mass index was 25.7 kg/m^2^. According to the American Society of Anesthesiologists (ASA) physical status classification, 76% of patients had severe systemic disease (ASA class >2). Most patients (91%) had an oncological indication for cystectomy, with non–organ-confined tumor growth (≥T3a) in 35% of cases. The median operation time was 214 min (IQR 178-271.5). The median length of hospital stay was 18 d (IQR 16-22). Major complications (Clavien-Dindo grade ≥IIIb) occurred in 16%, minor complications (grade I–IIIa) in 61%, and no complications in 23% of patients. The median CCI score was 22.6. The median leukocyte count was 10.2 × 10^9^ cells/l (IQR 8.5-11.9) and the median CRP level was 6.9 mg/dl (IQR 5.4-9.7) on POD1. IL-6 levels were elevated in 100% of patients (median 95.9 pg/ml, IQR 56.0-174.0) and ranged from 12 to 2319 pg/ml. PCT levels were elevated in 96% of patients (median 0.4 ng/ml, IQR 0.2-0.9) and ranged from <0.1 to 15.7 ng/ml (Table 1 and Fig. 1).

**Table 1:** Clinicopathological characteristics and infection markers on the first postoperative day in the overall cohort of patients undergoing open cystectomy and by urinary diversion group

	Overall cohort *n* = 186 (100%)	Ileal conduit *n* = 122 (66%)	Ileal NB *n* = 64 (34%)	*p* value
Median age, yr (IQR)	71.6 (64.8-79.3)	75.0 (68.8-81.6)	65.5 (61.4-71.5)	**<0.001**
Sex, *n* (%)				0.461
Male	148/186 (80)	99/122 (81)	49/64 (77)	
Female	38/186 (20)	23/122 (19)	15/64 (23)	
Median body mass index, kg/m^2^ (IQR)	25.7 (23.1-28.4)	25.5 (23.2-28.0)	25.9 (22.6-29.4)	0.430
Diabetes, *n* (%)	32/186 (17)	21/122 (17)	11/64 (17)	0.996
ASA class>2, *n* (%)	142/186 (76)	94/122 (77)	48/64 (75)	0.755
Oncological indication for cystectomy, *n* (%)	169/186 (91)	106/122 (87)	63/64 (98)	**0.009**
Neoadjuvant chemotherapy, *n* (%)	33/186 (18)	22/122 (18)	11/64 (17)	0.923
Non–organ-confined tumor (≥T3a), *n* (%)	58/165 (35)	37/102 (36)	21/63 (33)	0.701
Median operation time, min (IQR)	214 (178-271.5)	221 (178.5-277)	206 (178-252)	0.259
Median hospital stay, d (IQR)	18 (16-22)	16.5 (15-22)	20 (18-22)	**0.005**
Complications by Clavien-Dindo grade, *n* (%)				0.096
Major (grade ≥IIIb)	29/186 (16)	24/122 (20)	5/64 (8)	
Minor (grade I–IIIa)	114/186 (61)	70/122 (57)	44/64 (69)	
None	43/186 (23)	28/122 (23)	15/64 (23)	
Infection markers on POD1				
Median leukocyte count, 10^9^ cells/l (IQR)	10.2 (8.5-11.9)	10.2 (8.2-12.0)	10.2 (8.9-11.8)	0.652
Median CRP, mg/dl (IQR)	6.9 (5.4-9.7)	7.0 (5.3-10.3)	6.6 (5.5-8.0)	0.122
Median IL-6, pg/ml (IQR)	95.9 (56.0-174.0)	102.0 (58.0-191.5)	80.3 (53.2-149.3)	**0.021**
Median PCT, ng/ml (IQR)	0.4 (0.2-0.9)	0.5 (0.2-0.9)	0.3 (0.2-0.5)	0.357
Positive WDF culture, *n* (%)	20/160 (13%)	13/102 (13%)	7/58 (12%)	0.901

NB = neobladder; ASA = American Society of Anesthesiologists; POD1 = postoperative day 1; CRP = C-reactive protein; IL-6 = interleukin-6; PCT = procalcitonin; WDF = wound drainage fluid.

**Fig. 2:**
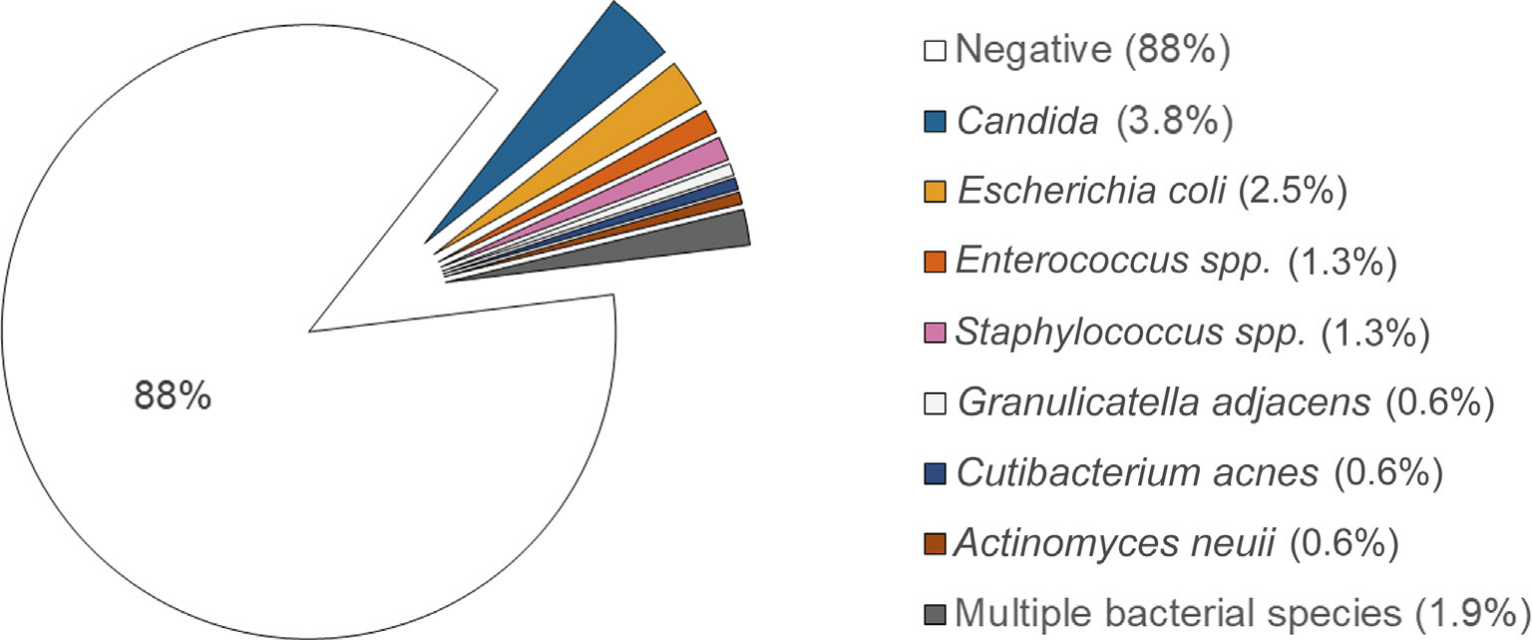
Microbial species detected in wound drainage fluid on the first postoperative day after cystectomy.

Microbial growth in WDF on POD1 was detected in 20/160 cases (13%). Fig. 2 shows the microbial diversity observed. *Candida* was identified in six cases (3.8%). *Escherichia coli* in four cases (2.5%), and *Enterococcus* and *Staphylococcus* spp. in two cases each (1.3%). *Granulicatella adjacens*, *Cutibacterium acnes*, and *Actinomyces neuii* were each detected once. In 3/160 cases (1.9%), multiple bacterial species were detected, including *Enterococcus* spp. in all three cases.

### 3.2 Incidence of in-hospital infectious complications after cystectomy

During their inpatient stay, 47% of the patients developed fever (≥38.0 °C) and 44% received intravenous antibiotic therapy in addition to the hospital standard protocol. The median time until initiation of first intravenous extension of antibiotic therapy was 6 d (IQR 8) after surgery. The additional intravenous antibiotics most frequently used were piperacillin + tazobactam (49%) and meropenem (23%). In 13% of all cases (24 of 186 patients), more than one additional intravenous antibiotic was given. Wound infections were identified in 17 patients (9.1%). The predominant pathogens found in wound cultures were *Enterococcus* spp. (41%) and *E. coli* (29%). Other infections were observed in nine patients (4.8%), including *Clostridium difficile*–associated diarrhea (3 cases), pneumonia (2 cases), COVID-19 (2 cases), port infection (1 case), and liver abscess (1 case).Positive blood cultures were found in eight patients (4.3%; Table 2).

**Table 2:** Incidence of postoperative infectious complications in the overall cohort of patients undergoing open cystectomy and by urinary diversion group

Parameter	Overall cohort *n* = 186 (100%)	Ileal conduit *n* = 122 (66%)	Ileal neobladder *n* = 64 (34%)	*p* value
Fever ≥38.0 °C, *n* (%)	87/186 (47)	56/122 (46)	31/64 (48)	0.819
Additional IV antibiotic therapy, *n* (%)	81/186 (44)	57/122 (47)	24/64 (38)	0.228
Wound infection, *n* (%)	17/186 (9.1)	11/122 (9.0)	6/64 (9.4)	0.936
Other infection, *n* (%)	9/186 (4.8)	8/122 (6.6)	1/64 (1.6)	0.267
Positive blood culture, *n* (%)	8/186 (4.3)	7/122 (5.7)	1/64 (1.6)	0.265
Median CCI (IQR)	22.6 (8.7-29.6)	22.6 (8.7-33.95)	20.9 (8.7-22.6)	0.150
Median IMC/ICU nights (IQR)	1 (0-1)	1 (0-1)	0 (0-1)	0.095

CCI = Comprehensive Complication Index; IMC/ICU = intermediate care/intensive care unit; IQR = interquartile range; IV = intravenous.

The most prevalent pathogens identified were *Enterobacter cloacae*, *Enterococcus faecium*, and *Klebsiella pneumoniae*, each detected in 25% of patients (2 out of 8). *E. coli* and *Candida albicans* were each detected in one case.

### 3.3 Comparison of IC and NB groups

Median age was significantly lower in the NB group than in the IC group (65.5 vs 75.0 yr; *p* < 0.001), and an oncological indication for cystectomy was more frequent in the NB group (98% vs 87%; *p* = 0.009). There were no significant differences between the IC and NB groups in sex distribution, body mass index, ASA class, rate of neoadjuvant chemotherapy, local tumor stage, or operation time. Median hospital stay was longer for the NB group than for the IC group (20 vs 16.5 d; *p* = 0.005), but there was no significant difference in the median number of IMC/ICU nights between the IC and NB groups. On POD1, median IL-6 was significantly higher in the IC group (102.0 vs 80.3 pg/ml; *p* = 0.021), but there were no significant differences in leukocyte count or CRP or PCT levels between the IC and NB groups. Similarly, the positive WDF culture rate did not significantly differ (IC 13% vs NB 12%; *p* = 0.901). We also found no significant differences in the incidence of postoperative fever, Clavien-Dindo complications by grade, or CCI scores (Table 1 and Table 2).

### 3.4 Evaluation of IL-6, PCT, and WDF culture positivity as early indicators of infectious complications

#### 3.4.1 IL-6

There was no difference in IL-6 levels on POD1 between the subgroups with and without fever. Median IL-6 was significantly higher in the group that received additional intravenous antibiotic therapy (121.5 vs 93.3 pg/ml; *p* = 0.033), but there was no significant difference in IL-6 levels between the subgroups with and without positive blood cultures. Spearman correlation analysis showed significant but low correlations between IL-6 levels and both Clavien-Dindo complication grade (r = 0.25, *p* = 0.001) and CCI score (r = 0.23, *p* = 0.002). Correlation between IL-6 and length of hospital stay was not statistically significant. Significant but low correlation was observed between IL-6 and the number of IMC/ICU nights (r = 0.31, *p* < 0.001; Table 3).

**Table 3:** Evaluation of interleukin 6, procalcitonin, and wound drainage fluid culture on postoperative day 1 as early markers for infectious complications

**Variable**	**Infectious complications**		*p* value
	Yes	No	
	**Median interleukin 6, pg/ml (IQR)**		
Fever ≥38.0 °C	92.7 (61.9-187.3)	95.9 (49.0-153.0)	0.155
Additional intravenous antibiotic therapy	121.5 (58.9-222.3)	93.3 (55.7-131.0)	**0.033**
Positive blood culture	69.8 (52.0-353.5)	95.85 (56.3-172.5)	0.341
Wound infection	187.0 (89.6-234.0)	91.15 (54.1-157.0)	0.631
Other infection	95.2 (158.7)	95.85 (110.78)	0.892
Clavien-Dindo complication grade	r = 0.25		**0.001**
Comprehensive Complication Index	r = 0.23		**0.002**
Hospital stay	r = 0.09		0.220
IMC/ICU nights	r = 0.31		**<0.001**
	**Median procalcitonin, ng/ml (IQR**)		
	Yes	No	*p* value
Fever ≥38.0 °C	0.40 (0.2-0.9)	0.40 (0.2-0.7)	0.546
Additional intravenous antibiotic therapy	0.40 (0.2-0.7)	0.30 (0.2-0.9)	0.273
Positive blood culture	0.40 (0.3-0.5)	0.40 (0.2-0.9)	0.848
Wound infection	0.50 (0.3-0.8)	0.30 (0.2-0.8)	0.427
Other infection	0.60 (0.3-1.5)	0.40 (0.2-0.8)	0.203
Clavien-Dindo complication grade	r = 0.13		0.112
Comprehensive Complication Index	r = 0.14		0.082
Hospital stay	r = −0.12		0.153
IMC/ICU nights	r = 0.21		**0.007**
	**Wound drainage fluid culture**		
	Positive	Negative	*p* value
Fever ≥38.0 °C, *n* (%)			0.786
Yes	10/75 (13)	65/75 (87)	
No	10/84 (12)	74/84 (88)	
Additional intravenous antibiotic therapy, *n* (%)			0.133
Yes	12/71 (17)	59/71 (83)	
No	8/89 (9)	81/89 (91)	
Positive blood culture, *n* (%)	1/20 (5)	3/140 (2.1)	0.417
Wound infection, *n* (%)	7/20 (35)	7/137 (5.1)	**<0.001**
Other infection, *n* (%)	0/19 (0)	7/139 (5)	0.599
Median Comprehensive Complication Index (IQR)	22.6 (7.38)	22.6 (22.55)	0.445
Median hospital stay, d (IQR)	20.5 (5.25)	18 (5.75)	0.998
Median IMC/ICU nights (IQR)	1 (0-1)	1 (0-1)	0.772

IMC/ICU = intermediate care/intensive care unit; IQR = interquartile range; r = Spearman’s rank correlation coefficient.

#### 3.4.2 PCT

There were no significant differences in PCT levels between subgroups with and without fever, subgroups with and without additional intravenous antibiotic therapy, or subgroups with and without positive blood cultures. There were no significant correlations between PCT levels and Clavien-Dindo complication grade, CCI score, or length of hospital stay. Significant but low correlation was observed between PCT and the number of IMC/ICU nights (r = 0.21, *p* = 0.007; Table 3).

#### 3.4.3 WDF culture positivity

There were no significant associations between WDF culture positivity and fever, additional antibiotic therapy, blood culture positivity, other infections, CCI scores, length of hospital stay, or IMC/ICU nights. The only infectious outcome parameter with a higher prevalence in the group with a positive WDF culture was the wound infection rate (35% vs 5.1%; *p* < 0.001; Table 3).

We also performed logistic regression analyses to examine the influence of clinicopathological characteristics and infection markers on postoperative fever, additional antibiotic therapy, and major Clavien-Dindo complications (Supplementary Table 1). No significant predictors for any of these outcomes were identified among the variables assessed. The incidence of the other infectious outcome parameters (wound infection, other infection, positive blood culture) was too low for multivariate analysis.

## 4 Discussion

### 4.1 Incidence of in-hospital infectious complications after cystectomy

We found a high incidence of infectious complications following open cystectomy. Nearly half of the patients developed fever or required additional intravenous antibiotic therapy beyond the standard institutional protocol of cefuroxime for 5 d and metronidazole for 3 d.

Despite the high rate of fever, only a small proportion of all patients had positive blood cultures. The incidence of infectious complications after cystectomy ranges from 5% to 40% [[1], [2], [3], [4]]. However, the term “infectious complication” is often not clearly defined. In a systematic review by Antonelli et al, the blood infection rate after cystectomy ranged from 2% to 19% [1].

### 4.2 Comparison of IC and NB groups

Patients in the NB group were generally younger and more likely to undergo cystectomy for oncological reasons than patients in the IC group, but there were no other significant differences in clinicopathological characteristics. Although median hospital stay was longer in the NB group, the number IMC/ICU nights did not significantly differ between the groups. We also found no significant differences in the incidence of postoperative fever or Clavien-Dindo complication grades, or in CCI scores. These findings suggest that although certain demographic and clinical characteristics differ between IC and NB groups, the postoperative infectious outcomes are largely comparable. Comparison to the literature is challenging owing to sparse in-hospital data focused on infectious complications. Incidence rates reported vary widely, ranging from 5% to 40% [[1], [2], [3], [4]]. A retrospective propensity score analysis of a US population-based cohort of 25 064 patients by Roghmann et al. [4] revealed that the NB group had slightly fewer overall complications than the IC group (51% vs 54%; *p* < 0.001). The authors found no differences in infectious complications between the IC and NB groups (5.8% vs 5.7%; *p* = 0.776) [4]. In our study, there were no significant differences in the overall complication rate between the IC and NB groups to Clavien-Dindo grades and CCI scores. We also found no significant differences in infectious outcome parameters between the IC and NB groups. Roghmann et al. [4] did not define the term “infectious complication”, which makes comparison to our study difficult.

### 4.3 Evaluation of IL-6, PCT, and WDF culture positivity as early indicators of infectious complications

Almost all patients in our study had significantly elevated IL-6 and PCT levels on POD1. There are very limited retrospective data available for these biomarkers in patients following cystectomy. In a retrospective study involving 54 patients who underwent open cystectomy by Horosz et al. [26] patients, median IL-6 on POD1 was significantly higher in the group who developed complications than in the group without complications(413.2 vs 250.2 pg/ml; *p* = 0.01). Median IL-6 on POD1 was significantly lower in our study (95.9 pg/ml). In a retrospective study by Ding et al. [27] including 306 patients undergoing laparoscopic radical cystectomy, median postoperative PCT levels ranged between 0.69 and 2.9 ng/ml at various unstandardized postoperative time points. These levels are significantly higher than the median of 0.4 ng/ml observed on POD1 in our study, which may be because of earlier measurement.

Our findings revealed either an absence of associations or only weak correlations between IL-6 and PCT and the clinical outcome parameters in our study. It has been shown that both IL-6 and PCT are useful markers for diagnosis of sepsis [28,29]. However, measurement of these markers on POD1 does not appear to be helpful in predicting in-hospital infectious complications after cystectomy. Despite being elevated, IL-6 and PCT levels lacked predictive value for infectious complications in our study cohort. One possible explanation is that major surgery induces a significant rise in IL-6 and PCT levels as part of the systemic inflammatory response, and the extent of this rise correlates with the severity of the surgical trauma [30,31].

WDF cultures have not been analyzed in urological surgery before. Microbial growth was detected in 13% of cases in our study and involved a diverse spectrum of bacteria and *Candida* species. In visceral surgery, positive drainage culture rates ranged from 13% to 34% [18,19,21]. Similar to our findings, these previous studies also revealed high diversity of bacteria and *Candida* species, often with more than one organism involved [18,19,21]. A meta-analysis including 1288 patients with drainage cultures from six studies concluded that WDF cultures were not relevant in predicting surgical site infections due to their low positive predictive values [20]. However, most of these studies were carried out in orthopedic surgery. In our study, patients with a positive WDF culture had significantly higher risk of developing a wound infection, but we found no other associations with in-hospital infectious complications.

### 4.4 Limitations

Despite the prospective design, our study has some limitations. It was a single-center analysis, focusing only on an open surgical approach. Minimally invasive approaches (such as robot-assisted cystectomy) are increasingly common and could potentially influence postoperative outcomes, including the rates and severity of infectious complications. Furthermore, the suitability of IL-6, PCT, and WDF culture positivity as early indicators of postoperative infectious complications was only assessed on POD1. This may not accurately capture the trajectory of infectious complications, which can develop or escalate beyond this initial period. This narrow window potentially overlooks significant trends and limits the utility of these markers in predicting complications that may arise later in the postoperative course. Later measurements might show different results. In addition, lack of a comparison group of patients treated with different surgical techniques or postoperative management protocols precludes comprehensive analysis of the predictive capabilities of the biomarkers evaluated.

## 5 Conclusions

This is the first prospective study to focus primarily on infectious complications after cystectomy. We observed unexpectedly high incidence of clinically significant in-hospital infections: nearly half of the patients developed postoperative fever and required additional intravenous antibiotics. Of note, there were no significant differences in the rate of in-hospital infectious complications between the IC and NB groups. There was no correlation for IL-6 or PCT levels or WDF culture results with infectious complications in our cystectomy cohort. Future studies should evaluate the predictive properties of these measures at later time points (eg, POD3) and compare the incidence of postoperative infectious complications between open cystectomy and minimally invasive approaches such as robot-assisted cystectomy.

  ***Author contributions***: Benedikt Ebner had full access to all the data in the study and takes responsibility for the integrity of the data and the accuracy of the data analysis.

  *Study concept and design*: Ebner, Schulz.

*Acquisition of data*: Hirsch, Holz.

*Analysis and interpretation of data*: Ebner, Volz, Schulz.

*Drafting of the manuscript*: Ebner.

*Critical revision of the manuscript for important intellectual content*: Volz, Eismann, Hermans, Pyrgidis, Kidess, Semmler, Brinkmann, Aydogdu, Chaloupka, Apfelbeck, Lindner, Weinhold, Stief.

*Statistical analysis*: Ebner.

*Obtaining funding*: None.

*Administrative, technical, or material support*: None.

*Supervision*: Stief.

*Other*: None.

  ***Financial disclosures:*** Benedikt Ebner certifies that all conflicts of interest, including specific financial interests and relationships and affiliations relevant to the subject matter or materials discussed in the manuscript (eg, employment/affiliation, grants or funding, consultancies, honoraria, stock ownership or options, expert testimony, royalties, or patents filed, received, or pending), are the following: None.

  ***Funding/Support and role of the sponsor*:** None.

  ***Ethics statement*:** This study was conducted in accordance with the ethical standards of the Declaration of Helsinki, was approved by the university ethics committee (project number 18-059), and is registered on ClinicalTrials.gov as NCT05153694.

  ***Use of generative AI and AI-assisted technologies*:** During the preparation of this manuscript, the authors used Microsoft Copilot, Claude, and DeepL to enhance the fluency of the text and to perform grammar checks. After using these tools, the authors reviewed and edited the content as needed and take full responsibility for the content of the publication.
