# Supplementary material for: Prospective Evaluation of Blood-based and Microbiological Early Indicators of In-hospital Infectious Complications After Open Cystectomy
Source: Eur Urol Open Sci. 2025 Nov 18;83:1–8. doi: 10.1016/j.euros.2025.10.019 (PMC12666572; doi:10.1016/j.euros.2025.10.019)
Supplement: Supplementary Data 1 [file mmc1.docx]

| **(A)** | **Fever** | **OR** | **95% CI** | ***p*-value** |
| --- | --- | --- | --- | --- |
|  | Age ≥71.6 years | 1.01 | 0.53 - 1.94 | 0.975 |
|  | Gender female | 0.91 | 0.41 - 1.99 | 0.810 |
|  | BMI ≥25.7 kg/m² | 1.58 | 0.84 - 2.98 | 0.158 |
|  | Diabetes | 1.12 | 0.50 - 2.54 | 0.784 |
|  | ASA 3-4 | 0.86 | 0.42 - 1.75 | 0.680 |
|  | Non-oncological indication | 0.87 | 0.29 - 2.57 | 0.800 |
|  | Neoadjuvant chemotherapy | 1.41 | 0.63 - 3.12 | 0.402 |
|  | Non-organ confined tumor | 0.80 | 0.41 - 1.58 | 0.528 |
|  | Operation time ≥214 min | 0.95 | 0.51 - 1.78 | 0.878 |
|  | Neobladder | 1.01 | 0.50 - 2.05 | 0.971 |
|  | IL-6 ≥95.9 pg/ml | 0.89 | 0.47 - 1.71 | 0.728 |
|  | PCT ≥0.4 ng/ml | 1.18 | 0.62 - 2.24 | 0.611 |
|  | Wound drain fluid culture positive | 1.15 | 0.44 - 3.03 | 0.771 |
|  |  |  |  |  |
| **(B)** | **Additional i.v. antibiotic therapy** | **OR** | **95% CI** | ***p*-value** |
|  | Age ≥71.6 years | 0.95 | 0.49 - 1.85 | 0.875 |
|  | Gender female | 0.83 | 0.36 - 1.88 | 0.650 |
|  | BMI ≥25.7 kg/m² | 1.08 | 0.56 - 2.06 | 0.821 |
|  | Diabetes | 1.63 | 0.71 - 3.72 | 0.249 |
|  | ASA 3-4 | 1.76 | 0.83 - 3.71 | 0.139 |
|  | Non-oncological indication | 1.29 | 0.43 - 3.90 | 0.651 |
|  | Neoadjuvant chemotherapy | 0.87 | 0.38 - 1.98 | 0.738 |
|  | Non-organ confined tumor | 1.10 | 0.55 - 2.19 | 0.785 |
|  | Operation time ≥214 min | 1.01 | 0.53 - 1.92 | 0.971 |
|  | Neobladder | 0.71 | 0.35 - 1.47 | 0.360 |
|  | IL-6 ≥95.9 pg/ml | 1.13 | 0.59 - 2.19 | 0.709 |
|  | PCT ≥0.4 ng/ml | 1.16 | 0.60 - 2.22 | 0.662 |
|  | Wound drain fluid culture positive | 1.91 | 0.71 - 5.15 | 0.201 |
|  |  |  |  |  |
| **(C)** | **Major Clavien-Dindo complications** | **OR** | **95% CI** | ***p*-value** |
|  | Age ≥71.6 years | 2.02 | 0.76 - 5.33 | 0.157 |
|  | Gender female | 0.29 | 0.06 - 1.44 | 0.129 |
|  | BMI ≥25.7 kg/m² | 0.56 | 0.21 - 1.51 | 0.253 |
|  | Diabetes | 2.12 | 0.73 - 6.11 | 0.166 |
|  | ASA 3-4 | 1.83 | 0.55 - 6.03 | 0.324 |
|  | Non-oncological indication | 0.22 | 0.02 - 1.92 | 0.169 |
|  | Neoadjuvant chemotherapy | 1.18 | 0.38 - 3.64 | 0.776 |
|  | Non-organ confined tumor | 1.07 | 0.41 - 2.80 | 0.889 |
|  | Operation time ≥214 min | 1.31 | 0.51 - 3.37 | 0.581 |
|  | Neobladder | 0.49 | 0.15 - 1.58 | 0.233 |
|  | IL-6 ≥95.9 pg/ml | 2.17 | 0.84 - 5.63 | 0.110 |
|  | PCT ≥0.4 ng/ml | 1.49 | 0.59 - 3.79 | 0.403 |
|  | Wound drain fluid culture positive | 1.24 | 0.33 - 4.71 | 0.747 |

**Supp. Table 1:** Logistic regression analysis for predictors of **(A)** postoperative fever **(B)** additional i.v. antibiotic therapy **(C)** major Clavien-Dindo complications. Abbreviations: BMI: body mass index. ASA: American Society of Anaesthesiologists. IL-6: interleukin-6. PCT: procalcitonin. For continuous variables, the median was used as cut-off.
